# Bio-Based Valorization of Lignin-Derived Phenolic Compounds: A Review

**DOI:** 10.3390/biom13050717

**Published:** 2023-04-22

**Authors:** Ludmila Martínková, Michal Grulich, Miroslav Pátek, Barbora Křístková, Margit Winkler

**Affiliations:** 1 Institute of Microbiology of the Czech Academy of Sciences, Vídeňská 1083, 142 20 Prague, Czech Republic; 2 Faculty of Food and Biochemical Technology, University of Chemistry and Technology, Prague, Technická 5, 166 28 Prague, Czech Republic; 3 Institute of Molecular Biotechnology, Faculty of Technical Chemistry, Chemical and Process Engineering, Biotechnology, Graz University of Technology, Petersgasse 14, 8010 Graz, Austria; 4 Austrian Center of Industrial Biotechnology GmbH, Krenngasse 37, 8010 Graz, Austria

**Keywords:** lignin, vanillin, guaiacol, eugenol, isoeugenol, *p*-coumaric acid, alkyl phenols, ferulic acid, biotransformation, valorization

## Abstract

Lignins are the most abundant biopolymers that consist of aromatic units. Lignins are obtained by fractionation of lignocellulose in the form of “technical lignins”. The depolymerization (conversion) of lignin and the treatment of depolymerized lignin are challenging processes due to the complexity and resistance of lignins. Progress toward mild work-up of lignins has been discussed in numerous reviews. The next step in the valorization of lignin is the conversion of lignin-based monomers, which are limited in number, into a wider range of bulk and fine chemicals. These reactions may need chemicals, catalysts, solvents, or energy from fossil resources. This is counterintuitive to green, sustainable chemistry. Therefore, in this review, we focus on biocatalyzed reactions of lignin monomers, e.g., vanillin, vanillic acid, syringaldehyde, guaiacols, (iso)eugenol, ferulic acid, *p*-coumaric acid, and alkylphenols. For each monomer, its production from lignin or lignocellulose is summarized, and, mainly, its biotransformations that provide useful chemicals are discussed. The technological maturity of these processes is characterized based on, e.g., scale, volumetric productivities, or isolated yields. The biocatalyzed reactions are compared with their chemically catalyzed counterparts if the latter are available.

## 1. Introduction

The chemical industry is largely dependent on fossil raw materials, the availability of which decreases as prices increase. Therefore, new processes are being sought to utilize renewable materials. Plant oils have been used to obtain (cyclo)aliphatic compounds, but renewable resources for phenolic compounds and other aromatic compounds are underutilized. The most abundant potential sources of these compounds are lignins, which are widespread as constituents of lignocellulose in plant cell walls. However, the resistance of lignins and their complexes with (hemi)celluloses to degradation limits the impact of their use for the production of valuable phenolic compounds [1,2,3].

Nevertheless, the recent innovations in this research area have the potential to make the biorefining of lignocellulose more profitable and sustainable [2,4,5].

**Scheme 1:**
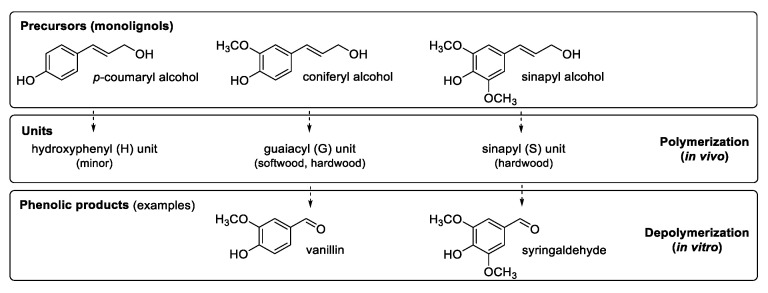
Precursors and units of lignin and selected phenolic compounds available by depolymerization of various lignin types [1,3].

The precursors of lignin [1,3,6] are 4-[(*E*)-3-hydroxyprop-1-enyl]phenol (synonyms: 4-hydroxycinnamyl alcohol, *p*-coumaryl alcohol) and its methoxy derivatives (coniferyl alcohol, sinapyl alcohol). Each of the three precursors (monolignols) gives a specific lignin unit: hydroxyphenyl (H), guaiacyl (G), or sinapyl (S). H-units are minor components, while G-units are widespread, and S-units are usually predominant in hardwood lignins. The unit type determines the type of product that can be obtained by depolymerization of a particular lignin (Scheme 1).

The sources of lignin are lignocellulosic materials (wood, herbaceous crops). Lignin can be separated from other components of lignocellulose (cellulose, hemicellulose) by a number of processes—kraft, sulfite, and soda pulping; acid-catalyzed hydrolysis; milling; extraction; etc. [3,6]. The type of process depends on the desired product, which can be either cellulosic material or lignin. The focus has been primarily on cellulosic materials for paper or biofuel production [3]. In these processes, lignin is usually burnt to meet the energy needs of the paper mill or biorefinery. The largest amounts of lignin are produced in the kraft process, which is used to make pulp and paper. However, this lignin is largely used in the form of “black liquor” (with hemicelluloses) as fuel, and only a small part of it is isolated. In addition, kraft lignin is poorly soluble and can be difficult to depolymerize, although some suitable methods (acid catalyzed, solvolytic) exist [3]. Nevertheless, kraft lignin can be used, e.g., to prepare cultivation media for the microbial production of lipids, polyhydroxyalkanoates, or methane [6].

Generally, lignins obtained by separation of cellulosic polymers are referred to as “technical lignins”, and are structurally different from native lignins. Milder methods of lignocellulosic fractionation are promising because the structure of the lignin thus obtained is similar to that of native lignin and, therefore, less resistant to depolymerizing agents (see below) [6]. For example, a promising approach to obtaining lignin is the “OrganoCat process”, i.e., oxalic acid-catalyzed hydrolysis of hemicellulose (140 °C, CO_2_, 1–2 MPa) combined with lignin extraction into a bio-based organic solvent—2-methyltetrahydrofuran [4].

Thus, the use of lignin to produce monomeric compounds requires depolymerization (“conversion”). A comprehensive review of depolymerization processes was recently published [6]. These processes are based on physical, chemical, physicochemical, or biological methods. With the exception of biological methods, they usually require high temperatures (200–800 °C) and chemicals or catalysts. The resulting mixtures are typically complex, and their fractionation is difficult. However, alternative types of lignin, e.g., lignin extracted from lignocellulose with solvents (organosolv lignin), can be depolymerized by milder means, including by enzymes [6,7]. For example, the lignin obtained by the above “OrganoCat process” can further be oxidized by laccase and then treated with 5 M NaOH at 37 °C to yield vanillic acid as the major product and smaller amounts of other phenolic compounds, such as 3,4-dimethoxybenzoic acid and 3,4-dihydroxycinnamic acid [5].

The simplification of depolymerization mixtures is referred to as “upgrading” and is largely based on chemical methods, although biological alternatives also exist [3].

The number of major low-molecular-weight compounds formed during the depolymerization of lignin is limited. The type of product depends on the lignin structure (see above) and on the depolymerization method (e.g., chemicals, pH, temperature). There are two main types of products—methoxyphenols (2-methoxyphenol and its derivatives), and phenols. These compounds can be used to produce other chemicals. So far, the reduction of phenols and 2-methoxyphenol (guaiacol) to phenol [8], cyclohexanols [9,10], or cyclohexanes [11,12] represents the most extensively studied examples. For instance, cyclohexanols are important precursors for fine and pharmaceutical chemicals [9], as well as for polymer building blocks (e.g., caprolactam, adipic acid) [3].

In summary, the conversion of lignocellulose to valuable aromatic compounds involves the fractionation of lignocellulose, depolymerization of lignin, upgrading, and, optionally, follow-up reactions. These steps largely require fossil resources. However, this is contrary to the concept of sustainable processing and circular economy, and compromises the benefits of the abundant and renewable lignin. Therefore, it is an attractive option to process lignin using biological catalysts. This will also reduce energy consumption due to the mild reaction conditions.

In the past few years, a number of reviews have addressed various general aspects of lignin valorization, such as lignin pretreatment [13] and depolymerization [3,13,14,15,16,17], upgrading of depolymerized lignins [3,14], and types of lignin-based products [6,15,18]. In addition, specialized reviews have focused on lignin pyrolysis [19], lignin reactions catalyzed by cytochrome P450 enzymes [2], the production and uses of vanillin [1,20,21], biotransformations of phenolic acids [22,23], and lignin-derived medical materials [24].

This review differs from those mentioned above in timescale and focus. We have been especially interested in the biotransformation of phenolic compounds directly obtained by the depolymerization of lignin. We have characterized these reactions in terms of the catalyst, conversion, selectivity, isolated yield, and/or volumetric productivity, if data are available. Attention was also paid to the efficiency and technological maturity of the proposed processes. In addition, we compared the biocatalyzed reactions to chemical processes, if the latter existed. This review is based on original research largely published between 2017 and 2023.

## 2. Vanillin

**Scheme 2:**
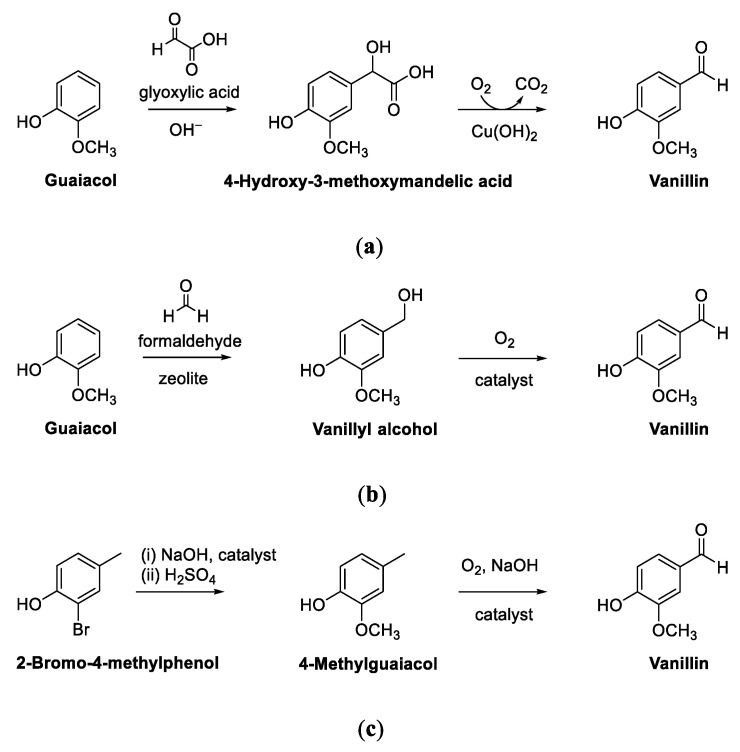
Industrial production of vanillin from guaiacol via (**a**) the Riedel process, (**b**) the Solvay process [1,25], and (**c**) from 2-bromo-4-methylphenol [25].

Vanillin (4-hydroxy-3-methoxybenzaldehyde) is a highly sought-after product with a yearly production of about 20,000 tons, according to data from 2014. Natural vanillin is prepared from its plant producers (genus *Vanilla*). It is very expensive, and covers less than 1% of the demand for vanillin. Artificial vanillin (“chemical vanillin”) is largely derived from fossil materials (usually from guaiacol obtained from phenol), with only about 15% produced from wood. The chemical processes consist of several steps and require reagents (glyoxylic acid or formaldehyde), organic solvents, and metal catalysts. The well-known Riedel process (Scheme 2a) suffers from a relatively low conversion rate (≈74%) in the guaiacol–glyoxylic acid condensation step, probably due to side reactions of glyoxylic acid. An innovative Solvay process (Scheme 2b) replaced this step with a guaiacol–formaldehyde reaction to vanillyl alcohol followed by oxidation to vanillin [1]. In addition, an emerging chemical process starts from 2-bromo-4-methyl phenol (BMP) (Scheme 2c) [25]. The industrial and innovative processes were compared in terms of their environmental impact. The traditional guaiacol-based process involves a number of disadvantages (high demands for energy, health risks, waste, etc.), and although the BMP-based process does not fully eliminate them, the amount of waste is lower [25].

Biocatalyzed processes use other substrates, such as vanillic acid, ferulic acid, and isoeugenol (see below). This opens up possibilities to replace “chemical vanillins” with “biovanillins”. Biological processes for the de novo synthesis of vanillin from sugars have also been proposed [1]. Nevertheless, the environmental impact and economic viability of the biobased methods must also be carefully analyzed with respect to electricity, ultrapure water consumption, etc. [25].

Natural and artificial vanillin, as well as the synthetic analog ethyl vanillin, are used as aromas in foods and pharmaceuticals and as fragrances in household products and cosmetics. Vanillin is also a promising synthetic intermediate for pharmaceuticals (e.g., L-3,4-dihydroxyphenylalanine for the treatment of parkinsonism) [1] and polymers [26,27,28]. In addition, new compounds with vanillin cores have been synthesized that are active against some plant bacteria and viruses [29], or that show indications of positive effects to be used in the treatment of Alzheimer’s disease [30]. Vanillin by itself was found to show anticancer activity [31], and to support the action of doxorubicin [32].

The production of vanillin from lignin was introduced in the 1930s. However, it was later abandoned by most producers due to environmental concerns (discharge of alkaline wastewater), limited lignin supply, and competition with petroleum-based vanillin production. Nevertheless, this type of process has been continued by the Borregaard company in Norway. The process starts with softwood (spruce) biomass and consists of sulfite pulping, oxidative depolymerization of the resulting lignosulfonate, and separation of vanillin (the major product) from byproducts (vanillic acid, acetovanillone, etc.) [1].

### Reduction of Vanillin to Vanillyl Alcohol

Whole cells of the yeast *Cystobasidium laryngis* reduced vanillin to vanillyl alcohol (4-hydroxy-3-methoxybenzyl alcohol) [33]. Vanillyl alcohol can be useful, e.g., for the production of new polymers [34] and, similarly to vanillin, it has wide therapeutic potential [35]. It is much more expensive than vanillin. The reduction of vanillin to vanillyl alcohol was previously carried out using a metal catalyst (Pd, Pt, Au) under a hydrogen atmosphere; this reaction was also applicable to 4-hydroxybenzaldehyde and syringaldehyde [36].

**Scheme 3:**
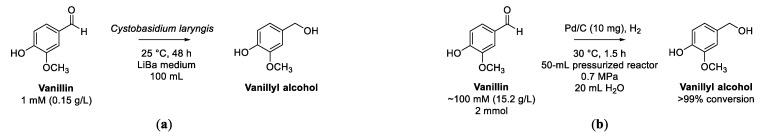
Transformation of vanillin to vanillyl alcohol (**a**) by whole cells of *Cystobasidium laryngis* [33] (the product was confirmed by UHPLC-MS and GC-MS, but not quantified); (**b**) the same reaction, catalyzed by Pd/C under an H_2_ atmosphere [36]. LiBa: Lilly–Barnett mineral medium containing d-glucose and l-asparagin [37].

The enzymatic reaction lags behind the chemical reaction in terms of concentration and reaction time (Scheme 3). Nevertheless, the possibility of performing the process at ambient pressure and aerobic conditions is attractive.

The enzymatic reduction of vanillin to vanillyl alcohol is also important in the context of reducing the negative impact of vanillin on bacteria, e.g., in biofuel production. The aldehyde functional group in vanillin is thought to exhibit toxic effects on living cells. Hence, the presence of vanillin is problematic in the context of bioethanol production. Its conversion to vanillyl alcohol reduces the toxicity of the lignocellulosic material to the microbes used in these processes [33].

## 3. Vanillic Acid

Vanillic acid (4-hydroxy-3-methoxybenzoic acid) is obtained as a byproduct of the depolymerization of lignosulfonates to vanillin. Its typical yields (0.5–1.5% and 0.2–2.4% from softwood and hardwood, respectively) are lower in comparison with vanillin (6–12% and 1–5% from softwood and hardwood, respectively) [1]. Separation of vanillic acid from its analogs was recently achieved by centrifugal partition chromatography [38]. In addition, the innovative OrganoCat process, followed by chemoenzymatic depolymerization of lignin (see above), provided vanillic acid at a 3.9% yield [5]. Vanillic acid is a potential precursor for building blocks of polyimides [39] or polyesters [40,41,42]. In addition, vanillic acid, per se, has potential therapeutical uses, as was summarized recently [43]. The bioreduction of vanillic acid to vanillin can be used to produce vanillin (Section 3.1), but also as the first step in the cascade reaction to vanillaldoxime and vanillonitrile (Section 3.2).

### 3.1. Reduction of Vanillic Acid to Vanillin

**Scheme 4:**
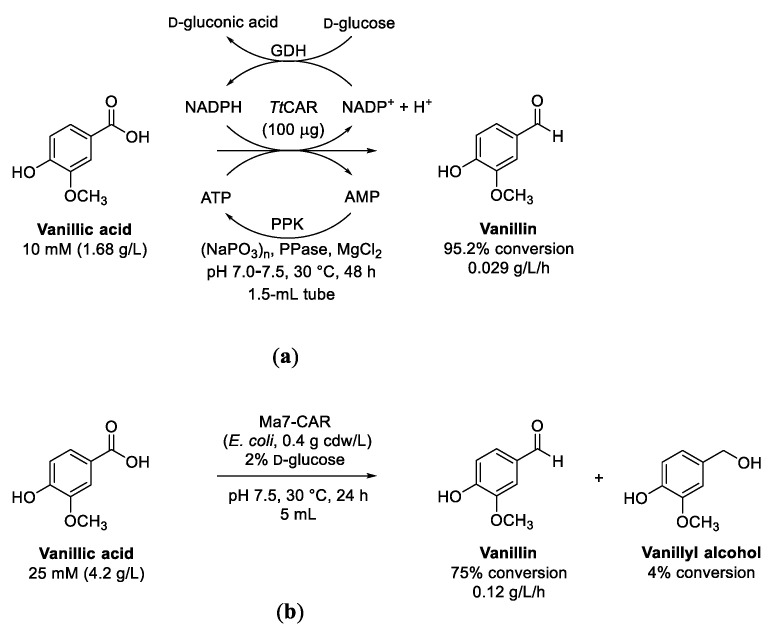
Reduction of vanillic acid to vanillin by carboxylate reductase (CAR) from (**a**) *Thermothelomyces thermophila* (*Tt*CAR; purified) [44] or (**b**) *Mycobacterium abscessus* (Ma7-CAR; whole-cell catalyst) [47]. cdw, cell dry weight; GDH, glucose dehydrogenase; PPK, polyphosphate kinase; PPase, pyrophosphatase.

Vanillic acid was converted to vanillin by carboxylic acid reductases (CARs; EC 1.2.1.30) [44,45,46,47]. The process was studied in more detail using CARs from *Thermothelomyces thermophila* [44] and *Mycobacterium abscessus* [47] (Scheme 4). This reaction can be carried out by purified CARs, which, however, require expensive cofactors (e.g., ATP, NADPH). However, an in vitro recycling of both cofactors has, meanwhile, become well-established [48]. This was demonstrated for vanillin production in a cell-free system containing the CAR from *T. thermophila* and auxiliary enzymes [44] (Scheme 4a). Alternatively, cellular metabolism can be hitchhiked. This approach has been illustrated using whole cells producing a CAR from *M. abscessus* for the same reaction (Scheme 4b). Over-reduction of vanillin to vanillyl alcohol may occur in whole-cell systems, but can be suppressed by increasing the substrate concentration (although at the expense of the reaction rate). Thus, the conversion rates of vanillic acid to vanillyl alcohol at 10 mM, 25 mM, and 50 mM of substrate were about 7%, 4%, and 0.1%, respectively, but the conversion rates to vanillin decreased from 92% to 75% and 34%, respectively [47].

### 3.2. Cascade Reactions from Vanillic Acid to Vanillaldoxime and Vanillonitrile

**Scheme 5:**
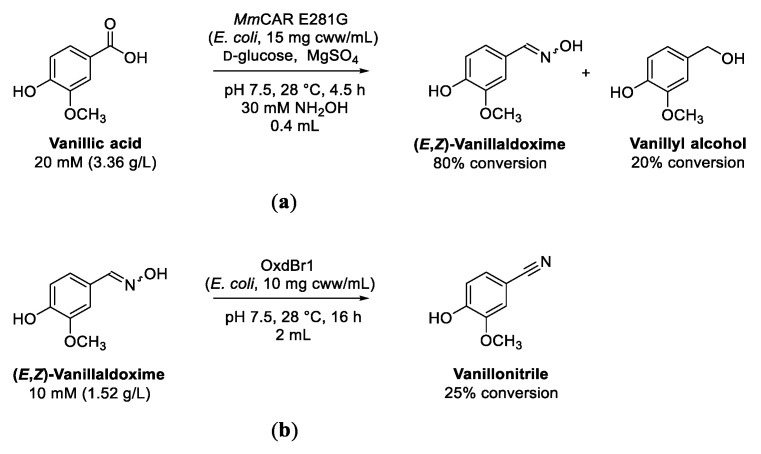
(**a**) Chemoenzymatic transformations of vanillic acid to (*E*,*Z*)-vanillaldoxime by carboxylate reductase (CAR) from *Mycobacterium marinum* (mutant E281G) in the presence of hydroxylamine; and (**b**) dehydration of (*E*,*Z*)-vanillaldoxime to vanillonitrile by aldoxime dehydratase OxdBr1 from *Bradyrhizobium* sp. [49]. cww, cell wet weight.

Vanillic acid can be converted directly to vanillaldoxime (4-hydroxy-3-methoxybenzaldehyde oxime) when the bioreduction of vanillic acid by CAR is performed in the presence of hydroxylamine. This transformation was particularly effective with whole cells producing the CAR from *Mycobacterium marinum* (mutant E281G) with 80% conversion to aldoxime. Nevertheless, the alcohol byproduct was formed at 20% conversion [49] (Scheme 5a).

Aldoximes are useful for the enzymatic synthesis of nitriles (Scheme 5b). This reaction is catalyzed by aldoxime dehydratase (Oxd) (EC 4.8.1.2-4) and has advantages over conventional nitrile syntheses (it is cyanide-free and proceeds under mild conditions) [50]. However, Oxds accepting vanillaldoxime have been found to be rare. The only Oxd with some activity for this substrate was OxdBr1 from *Bradyrhizobium* sp. [49].

### 3.3. Cascade Reaction from Vanillic Acid or Vanillin to Methoxyhydroquinone

**Scheme 6:**
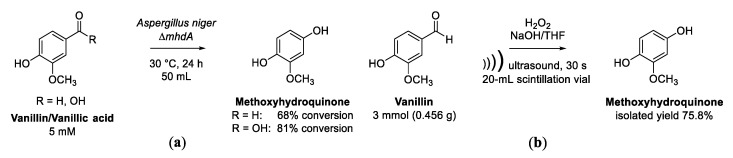
Production of methoxyhydroquinone (**a**) using *Aspergillus niger* mutant lacking the *mhdA* gene that encodes methoxyhydroquinone 1,2-dioxygenase [51], or (**b**) chemically [53]. THF, tetrahydrofuran.

The metabolism of vanillic acid in *Aspergillus niger* proceeds via methoxyhydroquinone (MHQ) and other intermediates to 3-oxoadipate, whereas the vanillic acid can be formed from vanillin, vanillyl alcohol, ferulic acid, veratric acid (3,4-dimethoxybenzoic acid), veratryl aldehyde, or veratryl alcohol. A deletion mutant lacking MHQ 1,2-dioxygenase was used for the production of MHQ from, e.g., vanillic acid or vanillin (Scheme 6a) [51]. MHQ has attractive uses in the production of biobased polymers [51] and redox batteries [52].

An alternative (chemical) production process of MHQ started from vanillin, which was oxidized using peroxide within short periods of time [53] (Scheme 6b). It seems that the biological process must be significantly improved to become competitive, as it has disadvantages such as its lower substrate concentration and longer reaction time. In addition, it was only demonstrated on an analytical scale [51]. Nevertheless, it may reduce the consumption of chemicals or increase the stability of MHQ due to mild conditions (MHQ is sensitive to pH and UV radiation [53]).

### 3.4. Cascade Reaction from Vanillic Acid to Gallic Acid

**Scheme 7:**
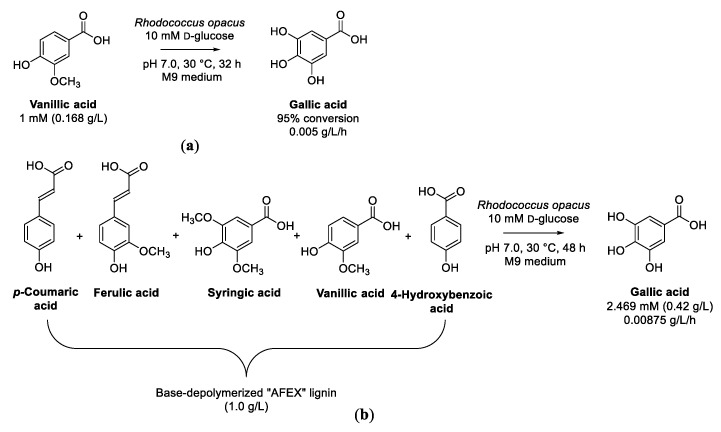
Biotransformations of (**a**) vanillic acid and (**b**) depolymerized lignin to gallic acid by engineered *Rhodococcus opacus* [54].

A recombinant organism for the production of gallic acid (3,4,5-trihydroxybenzoic acid) from vanillic acid (Scheme 7a) and other lignin-derived phenolics was obtained by engineering *Rhodococcus opacus* [54]. The resulting strain produced enzymes catalyzing demethylation and hydroxylation reactions, with protocatechuic acid (3,4-dihydroxybenzoic acid) as an intermediate. The genes for the demethylases were derived from *Sphingobium* sp., while the hydroxylase was endogenous (an improved mutant). The endogenous genes responsible for the catabolism of gallic acid were eliminated. A number of other phenolic acids (e.g., syringic, ferulic, *p*-coumaric, *p*-hydroxybenzoic) were transformed analogously. In addition, side-chain oxidation modules were incorporated that allowed aldehydes and alcohols to be converted into phenolic acids, and, further, to gallic acid, via the above route. Thus, the engineered strain was able to convert different phenolic substrates into the same product. Based on these achievements, a process was proposed for upgrading a mixture of phenolic compounds prepared by lignin depolymerization. The lignins used were the “ammonia fiber expansion” (AFEX) lignin (Scheme 7b) and corn stover [54]. The AFEX lignin results from biomass treatment with steam and ammonia vapor in a pressurized reactor [55]. It was treated with various enzymes ((hemi)cellulase, protease) prior to depolymerization, which was carried out with 2% NaOH at 120 °C for 40 min [54].

Gallic acid is a powerful antioxidant which naturally occurs as a building block of hydrolyzable tannins (a type of plant polyphenols), from which it is obtained by acid hydrolysis. It is promising for the synthesis of polymers and adhesives [56]. The medicinal potential of gallic acid, its analogs, and their hybrids with other pharmaceutical compounds has been reviewed [57]. These compounds can find applications in therapies for bacterial infections, cancers, diabetes, ulcers, etc. In addition, the gallic acid derivative octyl gallate, co-administered with ferulic acid, ameliorated the cognitive function in a murine model of Alzheimer´s disease [58].

## 4. Syringic Acid

Syringic acid (4-hydroxy-3,5-dimethoxybenzoic acid) is a product of the depolymerization of lignosulfonates made from hardwood. The typical yields of syringic acid (0.5–3.9%) are lower in comparison with other products, such as syringaldehyde (4–16%) or acetosyringone (1.5–4.2%) [1]. Syringic acid and its derivatives have many effects that are beneficial to health and potential uses in therapies for inflammation, infections, cancers, diabetes, hypertension, liver disorders, etc. Some edible plants and foodstuffs containing them (e.g., grapes, açaí palm berries, chard leaves, olives, radish, walnuts, honey, etc.) are good natural sources of syringic acid [59].

Syringic acid can be enzymatically reduced to syringaldehyde (Section 4.1). Another important compound resulting from the biotransformation of syringic acid is gallic acid (Section 3.4). Syringic acid is dimethyl ether of gallic acid.

### 4.1. Reduction of Syringic Acid to Syringaldehyde

**Scheme 8:**
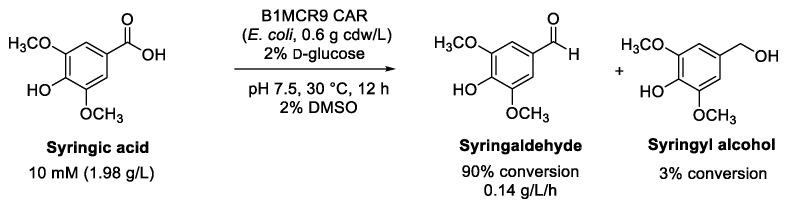
Biotransformation of syringic acid to syringaldehyde by carboxylate reductase (CAR) B1MCR9 from *Mycobacterium abscessus* [60].

CARs accepting syringic acid as a substrate were sought [60]. The enzyme suitable for the reduction of syringic acid to syringaldehyde (Scheme 8) was a CAR from *M. abscessus*. This is a different CAR than that used for the reduction of vanillic acid (Section 3.1; Scheme 4).

Syringaldehyde can also be obtained directly from hardwood lignin [1]. In addition, an organosolv lignin was transformed by laccase or cutinase to yield syringaldehyde, syringic acid, and 4-hydroxybenzoic acid as the main products in g/L concentrations [7]. Syringaldehyde is the starting material in the chemical reaction to syringaresinol, a bio-based alternative to bisphenol A [61]. The biological effects of syringaldehyde were recently summarized [62]. This review focused on the modeling of interactions between synringaldehyde and its molecular targets that are important in the treatment of diabetes and the alleviation of inflammation and oxidative stress.

## 5. Ferulic Acid

Ferulic acid ((2*E*)-3-(4-hydroxy-3-methoxyphenyl)prop-2-enoic acid, 4-hydroxy-3-methoxycinnamic acid) is produced with a yearly output of a few hundred tons; its main sources are the waste from the workup of sugar beets, sugarcane, and rice [1]. It forms ether and ester linkages to lignin and polysaccharides, respectively, in the plant cell wall. This strengthens the cell wall and restricts its growth [63]. The name ferulic acid is derived from the plant from which it was first obtained (genus *Ferula*) [64]. The interest in ferulic acid is due to its use in cosmetics [64] and its therapeutic potential in neurology [65,66], gastroenterology, and oncology [67].

The biotransformation of ferulic acid to vanillin has attracted much attention, as it is a potential path to a ”biovanillin”. Actually, ferulic acid is also the natural precursor of vanillin in *Vanilla* plants [21]. Ferulic acid can also be enzymatically transformed to vanillic acid [68] and synthetically important vinyl bromides [69].

### 5.1. Transformation of Ferulic Acid to Vanillin

**Scheme 9:**
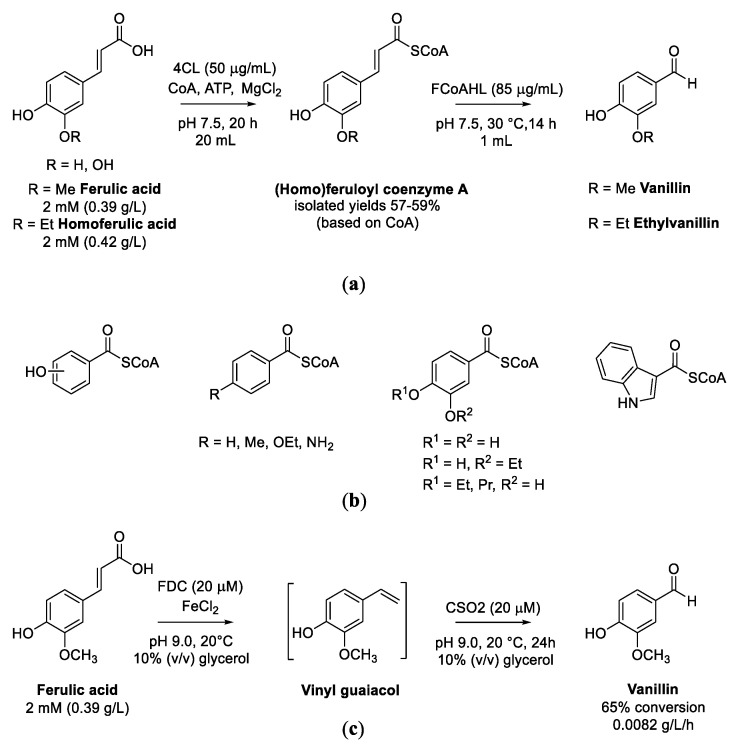
Enzymatic cascades from ferulic acid to vanillin or its analogs. (**a**) (Homo)ferulic acid to (ethyl)vanillin transformations using an artificial enzymatic cascade consisting of 4-coumarate:CoA ligase (4CL) and feruloyl-CoA hydratase/lyase (FCoAHL). Aliquots (1 mL) from the first step were used for the second step [72]. (**b**) Other CoA esters were produced analogously with isolated yields of 30–91% [72]. (**c**) Transformation of ferulic acid to vanillin by an artificial enzymatic cascade consisting of recombinantly produced and purified enzymes: ferulic acid decarboxylase (FDC) from *Bacillus pumilus* and an artificial variant of the carotenoid cleavage oxygenase (CSO2) from *Caulobacter segnis* [73].

The transformation of ferulic acid to vanillin occurs via feruloyl coenzyme A (feruloyl-CoA) [70,71,72] (Scheme 9a). The conjugation of ferulic acid and CoA is catalyzed by the 4-coumarate:CoA ligase (EC 6.2.1.12; 4CL). This enzyme accepts *p*-coumaric acid, ferulic acid, cinnamic acid, and analogs [72], and is also known as feruloyl-CoA synthetase [70]. The next step is catalyzed by feruloyl-CoA hydratase/lyase (EC 4.1.2.61, formerly EC 4.2.1.101; FCoAHL) to yield vanillin and acetyl-CoA [70,72].

An artificial cascade of this type was performed using 4CL from *Arabidopsis thaliana* and a bacterial FCoAHL, and this led to the production of vanillin and ethyl vanillin [72] (Scheme 8a). The first reaction of the cascade was also demonstrated with a number of other carboxylates (Scheme 9b).

A different type of pathway consisted of ferulic acid decarboxylation followed by oxygenation [73,74,75]. This artificial route does not require any cofactors and proceeds in one pot, which are significant advantages. The biocatalysts were ferulic acid decarboxylase (EC 4.1.1.102; FDC) and carotenoid cleavage oxygenase (EC 1.13.11.65; CSO2). They were overproduced in *E. coli* and used as enzymes in solution [73] or immobilizates (enzymes bound on a Sepabeads ion-exchange resin [75], or whole cells in alginate [74]). A conversion of up to 65% was obtained with an improved variant of CSO2 (Scheme 9c) [73].

**Scheme 10:**
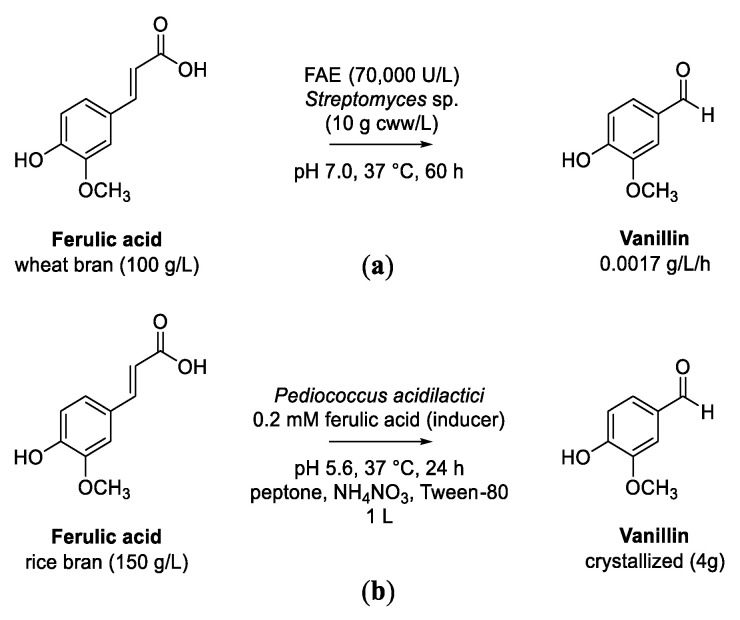
Transformation of (**a**) wheat bran [76] or (**b**) rice bran [78], as sources of ferulic acid, to vanillin by bacterial whole cells. Ferulic acid was liberated from wheat bran using ferulic acid esterase (FAE) from *Enterococcus lactis* [76]. cww, cell wet weight.

The transformation of ferulic acid to vanillin was also catalyzed by whole cells of various bacteria in flasks [76] or a 2/L bioreactor [77]. Whole cells of *Streptomyces* sp. produced vanillin from pure ferulic acid or wheat bran, a natural source of ferulic acid (Scheme 10a). The release of ferulic acid from wheat bran was supported by adding ferulic acid esterase [76]. Other sources of ferulic acid are rice bran and banana peels, which have been used for vanillin production using engineered *Pediococcus acidilactici* [78] or *Enterobacter hormaechei* [79], respectively. The former strain that heterologously expressed the genes encoding 4CL and FCoAHL, provided high vanillin yields (up to approximately 4 g crystallized vanillin per L) (Scheme 10b). It was chosen because of its ability to liberate ferulic acid from rice bran [78].

The processes catalyzed by whole cells of *Amycolatopsis* sp. (previously named *Streptomyces setonii*) [77] or recombinant *E. coli* [73,80] outperformed similar processes in terms of their volumetric productivity levels.

**Scheme 11:**
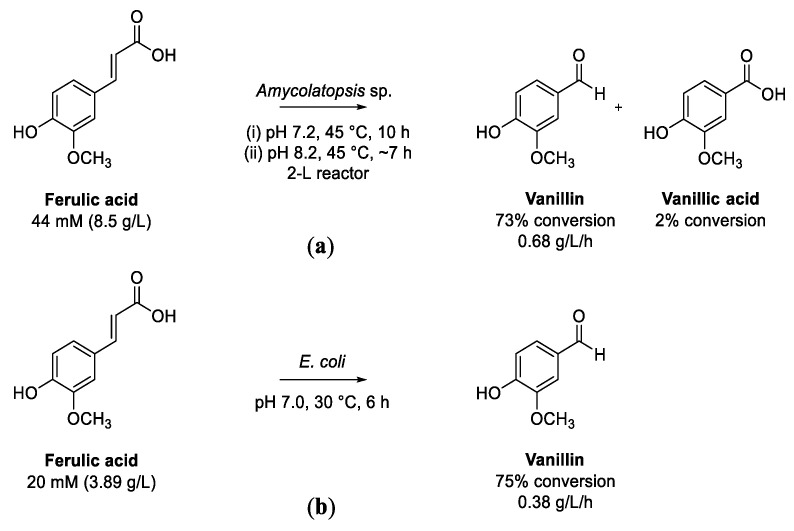
Transformation of ferulic acid to vanillin by whole cells of (**a**) *Amycolatopsis* sp. in a two-step process—(i) growth and (ii) production [77]—or (**b**) recombinant *E. coli* [71]. In (**a**), the biomass was collected by centrifugation and used for the next run. The average volumetric productivity of 7 runs was 0.46 g/L/h [77].

The disadvantage of the wild-type strain was the overoxidation of the product to vanillic acid [80] (Scheme 11a). This is caused by the tendency of the bacterium to detoxify vanillin (toxic for most bacteria). However, the production of vanillic acid can be alleviated by adding glucose and removing product [77]. The whole-cell system based on recombinant *E. coli* had comparable parameters, but without significant byproducts [73] (Scheme 11b). One of the highest conversions (94%) was achieved by combining computation and cultivations in flasks or bench-scale reactors, although for a substrate concentration (5 mM) [80] lower than that obtained in the above work (20 mM) [71].

Another *E. coli* strain carried the corresponding genes (from *Pseudomonas*) integrated into its chromosome. Ferulic acid was incorporated into agarose gel to avoid the inhibitory effects of high levels of this substrate. The ratio of vanillin to vanillyl alcohol (byproduct) was increased by process optimization (pH, phosphate concentration, etc.) [81].

### 5.2. Transformation of Ferulic Acid to Vanillic Acid

**Scheme 12:**
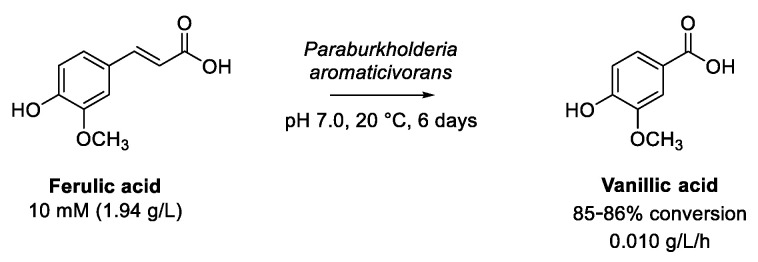
Transformation of ferulic acid to vanillic acid by a growing culture of *Paraburkholderia aromaticivorans* [68].

A soil isolate of *Paraburkholderia aromaticivorans* accumulated vanillic acid while growing on ferulic acid [68]. The transformation proceeded via vanillin. The pathway for the degradation of vanillic acid was missing. This resulted in a high yield of vanillic acid (Scheme 12). The strain also metabolized *p*-coumaric acid, *p*-hydroxybenzoic acid, and benzoic acid, but without accumulating products.

## 6. *p*-Coumaric Acid

*p*-Coumaric acid (*p*CA; synonyms: (2*E*)-3-(4-hydroxyphenyl)prop-2-enoic acid, 4-hydroxycinnamic acid) is obtainable, as a mixture with ferulic acid and vanillic acid, from herbaceous plants. The phenolic acids are released by alkaline treatment under relatively mild conditions (≤1 M NaOH, 100–160 °C) with a high yield (27% in total). This process is enabled by the prevalence of ester bonds (more labile to alkaline treatment than ether bonds) in the lignins of these plants [3]. Another source of *p*-CA and other phenolic acids is the waste from the processing of palm oil [23].

*p*-CA belongs to natural bioactive phenolic acids with great potential in nutraceuticals, pharmaceuticals, and cosmetics [23], and as building block in organic synthesis. For instance, recent works have demonstrated its use as a precursor of 4-hydroxybenzoic acid (*p*-HBA) (Section 6.1).

### 6.1. Transformation of p-Coumaric Acid to p-Hydroxybenzoic Acid

**Scheme 13:**
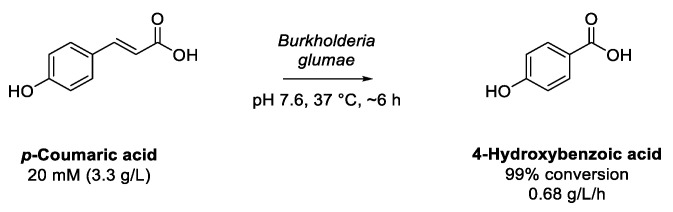
Transformation of *p*-coumaric acid to *p*-hydroxybenzoic acid by an engineered strain of *Burkholderia glumae* [82].

The shikimate pathway in *Burkholderia glumae* was engineered for the production of *p*-HBA from *p*CA. Two genes encoding enzymes that degrade *p*-HBA via the β-ketoadipate pathway—*p*-HBA-3-hydroxylase (EC 1.14.13.2) and benzoyl-CoA ligase (EC 6.2.1.25)—were deleted. In addition, the formation of the rate-limiting enzyme, *p*-hydroxycinnamoyl-CoA synthetase, was enhanced. This was achieved by using a strong *alk* promoter to express the corresponding gene, with dicyclopropylketone as an inducer. This organism converted 20 mM *p*CA to *p*-HBA with a 99% conversion rate [82] (Scheme 13).

The current production of *p*-HBA is based on petroleum-derived chemicals. *p*-HBA is a precursor for antimicrobial products with applications in pharmaceuticals and cosmetics (parabens) and for liquid crystal polymers [24,82]. It may be also used as a precursor for the production of bioactive glycosides arbutin (Section 6.1.1) and resveratrol. The use of *p*CA and other substrates for the synthesis of resveratrol has recently been reviewed [83]. In a de novo synthesis of gastrodin (4-hydroxymethylphenyl β-D-glucopyranoside) by engineered *E. coli*, *p*-HBA was utilized by the bacterium as an endogenous precursor. A CAR enzyme from *Nocardia,* along with endogenous alcohol dehydrogenases, reduced the *p*-HBA to alcohol. This strain also produced a glucosyltransferase from the plant *Rhodiola* [84]. Gastrodin is the key bioactive compound of the *Gastrodia elata* plant (orchid), traditionally used in Eastern medicine for the treatment of diseases of the central nervous system and circulatory system [84]. Recent studies (mostly preclinical) have confirmed its high potential for the treatment of, e.g., neurological disorders [85] and hypertension [86].

#### 6.1.1. Transformation of *p*-Hydroxybenzoic Acid to Arbutin

**Scheme 14:**
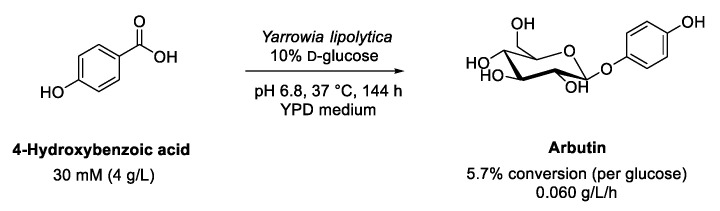
Transformation of *p*-hydroxybenzoic acid to arbutin by engineered *Yarrowia lipolytica* [88]. YPD, yeast–peptone–dextrose.

Arbutin (4-hydroxyphenyl β-D-glucopyranoside) occurs in plants (e.g., strawberry tree (*Arbutus unedo*), wheat, pear, bearberry). Herbal preparations containing it were known as antimicrobial medicines (against infections of the urinary tract) before the discovery of modern antibiotics [87]. Arbutin is also a known tyrosinase inhibitor and, therefore, a popular agent for skin whitening [88]. The biological process for its production employed an engineered strain of *Yarrowia lipolytica* (Scheme 14). This strain produced arbutin from *p*-HBA via hydroquinone; the reaction was catalyzed by 4-hydroxybenzoate 1-hydroxylase (EC 1.14.13.64). The glucosylation reaction was catalyzed by hydroquinone glucosyltransferase. The concentration of arbutin was up to 8.6 g/L, at which point the highest concentration of D-glucose (10%) was used [88].

## 7. Guaiacol and Alkyl Guaiacols

Recently, hydrothermal methods of lignin depolymerization (demonstrated on a gram scale) provided significant yields of guaiacol. The reactions proceeded at 300 °C, and the aromatics were extracted from the soluble fraction with toluene [89] or condensed by steam distillation [90]. The former method yielded guaiacol and 4-methylguaiacol (creosol) at a molar ratio of approximately 6:1 [89]. The latter yielded guaiacol and 2-methyl phenol (molar ratio 2:1) after condensation [90]. The pyrolysis of lignin at 275–350 °C yielded guaiacol with an even higher selectivity of up to 90.7% [91]. Moreover, guaiacol was the only product of lignin depolymerization catalyzed by a Lewis acid, lanthanum tris(trifluoromethanesulfonate), at 270 °C. This method yielded 0.32 g of guaiacol from 1.5 g of lignin [92]. 4-*n*-Propylguaiacol and other 4-alkylguaiacols were the major products of the reductive catalytic fractionation (RCF) of softwood lignin [93].

Guaiacol was used to chemically prepare phthalonitrile [94] and triphenol [26] for polymer synthesis. 4-*n*-Propylguaiacol aided in chemical syntheses of catechol [93] and γ-valerolactone, which is a compound suitable for the production of fuels and polymers [95].

*O*-Methylated lignin-derived compounds, including guaiacol and alkylguaiacols, are demethylated into catechols by cytochrome P450 monooxygenases (CYP) in microbes [96]. The CYP255A family consists of enzymes acting on guaiacol or alkylguaiacols. For example, the characterized CYP255A1 enzymes from rhodococci prefer 4-*n*-propylguaiacol as a substrate [2]. The CYP system can include ferredoxin and ferredoxin reductase, which transfer electrons from substrate to CYP [96]. The demethylation of guaiacol is the first step in the pathways to *cis*,*cis*-muconic acid (*c,c*-MA) or adipate (see below).

### Enzymatic Cascades from Guaiacol to cis,cis-Muconic Acid and Adipic Acid

*c,c*-MA is promising for the synthesis of bio-based chemicals (polymers, composite materials, fine chemicals, etc.) [90]. For instance, it can be converted to adipic acid, a monomer of nylon-6,6, by catalytic hydrogenation [97,98].

**Scheme 15:**
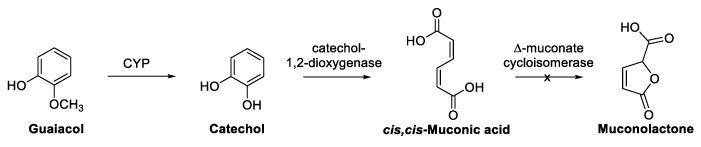
Proposed metabolism of guaiacol in a muconate isomerase-deficient mutant of *Amycolatopsis* sp. [90]. CYP, cytochrome P450 monooxygenase.

In the conversion process from guaiacol to *c,c*-MA, the demethylation product, catechol, was transformed by catechol-1,2-dioxygenase, encoded by the *catA* gene. The bacterium used (*Amycolatopsis* sp.) was a mutant with muconate cycloisomerase (EC 5.5.1.-) genes deleted, and thus accumulated *c,c*-MA [90] (Scheme 15).

**Scheme 16:**
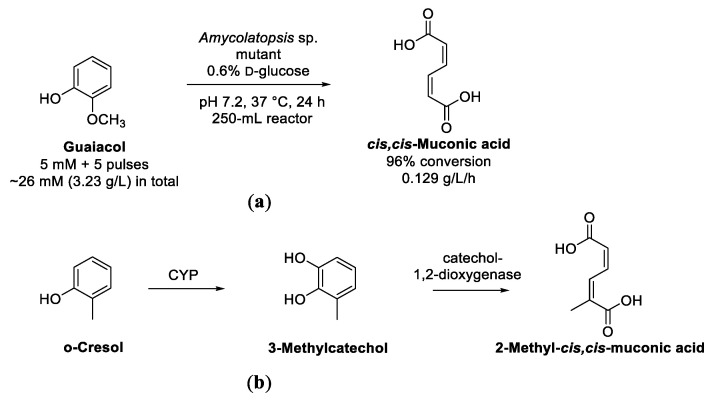
(**a**) Biotransformation of guaiacol to *cis*,*cis*-muconic acid (*c,c*-MA) by an *Amycolatopsis* sp. mutant unable to metabolize *c,c*-MA. (**b**) 2-Methyl-*c,c*-MA may be produced from 2-methylphenol in an analogous manner [90]. A hypothetical reaction mechanism is shown. CYP, cytochrome P450 monooxygenase.

The utility of this bacterium was demonstrated by a fed-batch conversion of guaiacol using a bench-scale reactor (Scheme 16a). *c,c*-MA was also produced by this mutant from a mixture of guaiacol (major product), 2-methylphenol (*o*-cresol), and phenol (minor product) obtained by hydrothermal depolymerization of lignin. An analogous process was proposed for the conversion of 2-methylphenol to 2-methyl-*c,c*-MA, which may serve as a new polymer building block [90] (Scheme 16b).

**Scheme 17:**
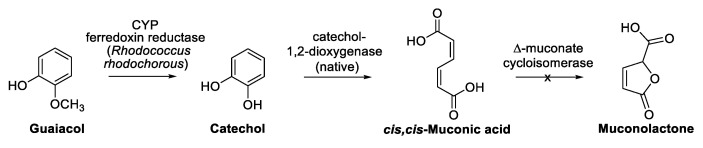
Metabolism of guaiacol in engineered *Pseudomonas putida* [99]. CYP, cytochrome P450 monooxygenase. The origin of each gene is in brackets.

In addition, the *Pseudomonas putida* strain, able to utilize guaiacol, was engineered for a similar process. Two genes participating in the transformation of *c,c*-MA were deleted. Moreover, genes coding for CYP and ferredoxin reductase (participating in the guaiacol demethylation) were introduced [99] (Scheme 17).

**Scheme 18:**
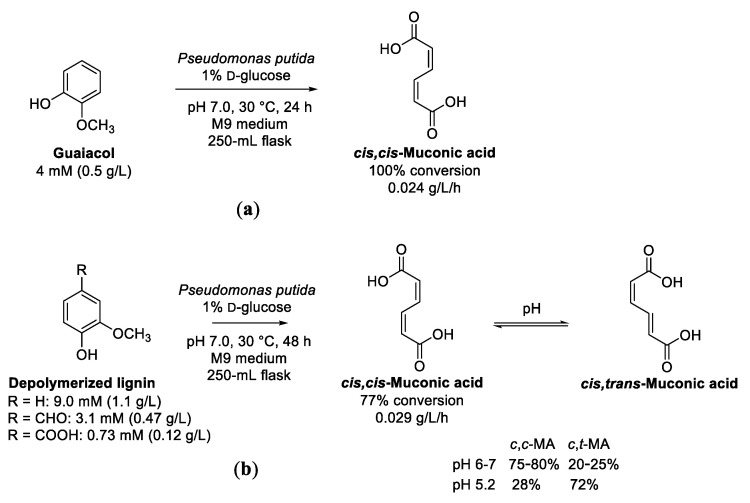
Biotransformations of (**a**) guaiacol or (**b**) a water-soluble fraction of depolymerized lignin to *cis*,*cis*-muconic acid (*c,c*-MA) by engineered *Pseudomonas putida. c,c*-MA is isomerized into *cis*,*trans*-muconic acid (*c,t*-MA) at an acidic pH level [99].

Guaiacol, as well as real or model fractions from lignin conversion (guaiacol, vanillin, vanillic acid) were used to prepare *c,c*-MA using this strain (Scheme 18a). The conversion of guaiacol was full in all cases. However, the product isomerized to the corresponding *cis*,*trans*-isomer if pH decreased during the run (Scheme 18b). Both isomers can be used for subsequent hydrogenation to adipic acid. However, if *c,c*-MA is the targeted product, the pH of the medium must be kept sufficiently high [99].

**Scheme 19:**
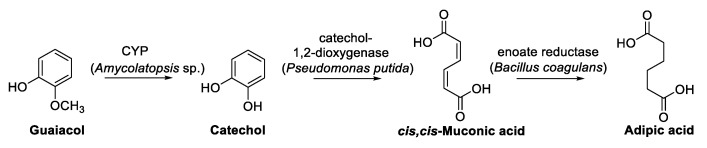
Metabolism of guaiacol by engineered *Escherichia coli* expressing genes for CYP, catechol-1,2-dioxygenase, and enoate reductase [97]. CYP, cytochrome P450 monooxygenase. The origin of each gene is in brackets.

An approach different from engineering native guaiacol degraders was the de novo construction of a guaiacol pathway in *E. coli*. The strain was tailor-made for a one-pot conversion of guaiacol to adipic acid. In addition to enzymes of the above pathway (CYP, catechol-1,2-dioxygenase), it also contained an enoate reductase (*Bc*ER) to reduce *c,c*-MA (Scheme 19), as well as molecular chaperones with positive effects on whole-cell activities.

**Scheme 20:**
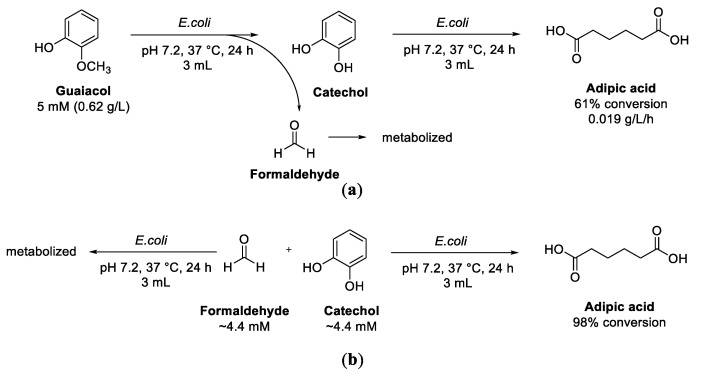
Biotransformations of (**a**) guaiacol or (**b**) catechol (in the presence of formaldehyde) to adipic acid by recombinant *Escherichia coli* [97]. The reactions proceeded via *cis*,*cis*-muconic acid (see Scheme 19). The strain heterologously expressed the genes for CYP, ferredoxin, catechol-1,2-dioxygenase, and enoate reductase.

The reaction of guaiacol to adipic acid proved to be feasible, although the conversion was medium (61% maximum) (Scheme 20a). This was caused by the low rate of the first reaction—the demethylation of guaiacol to catechol and formaldehyde. An analogous process starting from a mixture mimicking the products of guaiacol demethylation, i.e., catechol and formaldehyde (1:1), proceeded with an almost total conversion of catechol to adipic acid [97] (Scheme 20b).

## 8. Eugenol and Isoeugenol

Eugenol (2-methoxy-4-prop-2-enylphenol) and isoeugenol (2-methoxy-4-propenylphenol) are derivatives of guaiacol with propenyl side chains. The use of eugenol as a precursor for various building blocks of polymers was recently summarized [100]. Eugenol can be used to produce coatings [101,102] and composite materials [103]. Eugenol and especially isoeugenol are suitable for the biocatalyzed production of vanillin (see below).

### Oxidation of (Iso)Eugenol to Vanillin

**Scheme 21:**
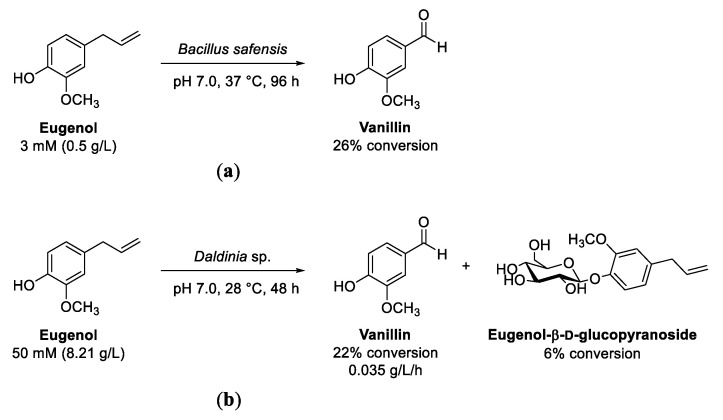
Transformation of eugenol to vanillin by whole cells of (**a**) *Bacillus safensis* [104] and (**b**) *Daldinia* sp. [105].

The transformation of eugenol by *Bacillus safensis* provided vanillin with a moderate conversion rate [104] (Scheme 21a). The plant-associated fungus *Daldinia* sp. transformed eugenol with a similar conversion rate, but at a concentration one order of magnitude higher [105]. Eugenol-β-D-glucopyranoside was found as a minor product (Scheme 21b).

**Scheme 22:**
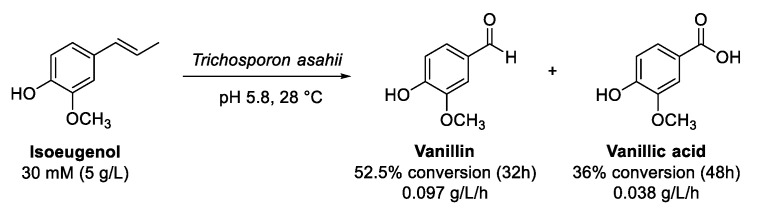
Transformation of isoeugenol to mixtures of vanillin and vanillic acid by *Trichosporon asahii* [106]. The maximum conversion for each product is shown.

Another fungus, *Trichosporon asahii* (yeast), transformed isoeugenol to vanillin and vanillic acid [106]. The conversions were higher than those obtained for eugenol in the aforementioned experiments—over 52% and 35% for vanillin and vanillic acid, respectively—and they were achieved after different reaction times (Scheme 22). The resulting products were mixtures of vanillin and vanillic acid at ratios depending on the reaction time (disregarding the residual isoeugenol). This may be a problem unless both products are streamed into a single one, as in the aforementioned production of *c*,*c*-MA from depolymerized lignin.

**Scheme 23:**
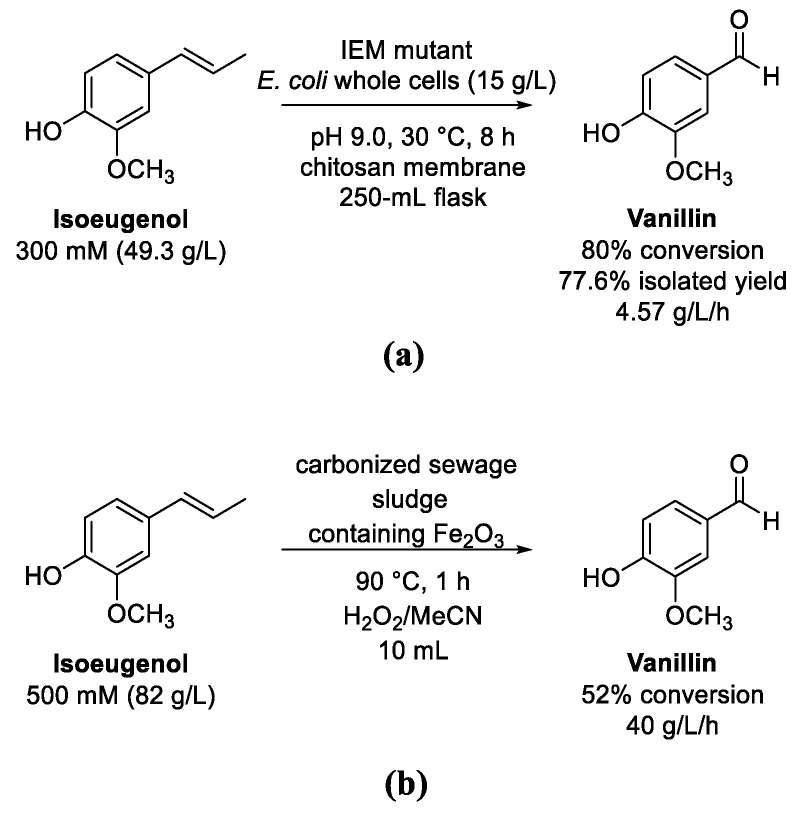
Transformation of isoeugenol to vanillin (**a**) by isoeugenol monooxygenase (IEM) from *Pseudomonas nitroreducens*. A mutant IEM (K83R/K95R/L273F) was used. The reaction mixture contained a chitosan membrane to withdraw vanillin, which was then released in 6% HCl (30 °C, 12 h) [107]. Alternatively, isoeugenol was (**b**) oxidized by H_2_O_2_ under catalysis of a biobased silica material with a high content of Fe^2+^ [111]. In (**b**), dimeric and oligomeric byproducts formed ≈35% of total product.

In contrast, engineered cells of *E. coli* carrying isoeugenol monooxygenase (EC 1.13.11.88; IEM) obtained by the expression of a gene from *Pseudomonas nitroreducens* [107] or from a metagenomic source [108] yielded only vanillin. In addition, the activity [108] and thermostability [107] of IEMs were improved by point mutations, the best variant being a triple mutant with approximately 3-fold to 25-fold increases in half-lives at 25–35 °C. This enzyme, in the form of whole cells, was used to prepare vanillin on a preparative scale (Scheme 23a), resulting in 1 g of purified product [107].

Alternatives of the above biocatalysts can be, e.g., cerium-containing zeolites [109], iron or cobalt salts on graphene oxide support [110], or carbonized sewage sludge containing high amounts of ferric oxide [111]. Although not a biocatalyst, the latter is derived from a renewable material, which makes the process sustainable. These catalysts transformed concentrations of isoeugenol much higher (e.g., 0.5 M, 82 g/L [110,111]) than the above biological processes and at shorter reaction times (Scheme 23b). Thus, the volumetric productivity levels were one order of magnitude higher than the best one achieved enzymatically. However, the main disadvantage of these methods is probably the formation of large amounts of byproducts, such as diphenylether [110], guaiacyl acetone and MHQ [109], or dimers and oligomers [111].

**Scheme 24:**
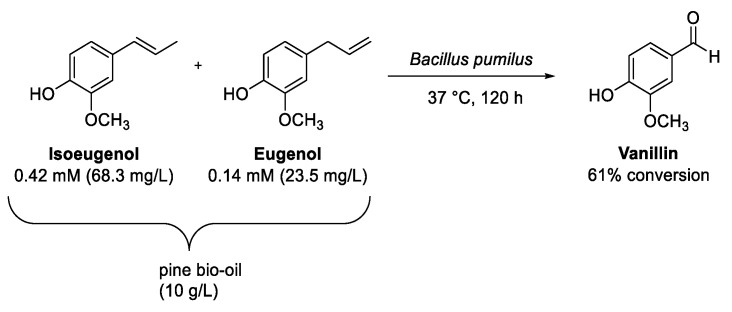
Upgrade of bio-oil from masson pine via transformation of isoeugenol and eugenol to vanillin by whole cells of *Bacillus pumilus* [112]. The bio-oil also contained vanillin (15.9 mg/L) prior to biotransformation. The final concentration of vanillin after biotransformation was 72.0 mg/L.

Biotransformations were also applied to the upgrade of pine bio-oil (Scheme 24). This material was obtained by the fast pyrolysis of softwood from masson pine. The oil contained multiple compounds, including vanillin, eugenol, and isoeugenol, in concentrations of tens of mg/L. The oxidation of isoeugenol and eugenol was catalyzed by whole cells of *Bacillus pumilus*. Isoeugenol was a much better substrate than eugenol, as determined from the biotransformations of standards. The activity of isoeugenol was confirmed in four monooxygenases of this strain [112].

## 9. Alkylphenols

4-Alkylphenols can be obtained from lignins by pyrolysis combined with partial defunctionalization (removal of methoxy group). The latter reaction is catalyzed by various metal catalysts (Pd/C, Fe/C, CoMo etc.) [3]. The aromatic and aliphatic C-H bonds in alkylphenols can be functionalized (hydroxylated) by tyrosinase (TYR; EC 1.14.18.1) (Section 9.1) and vanillyl alcohol oxidase (VAO; EC 1.1.3.38) (Section 9.2), respectively.

### 9.1. O-Hydroxylation of Alkylphenols to Catechols

**Scheme 25:**
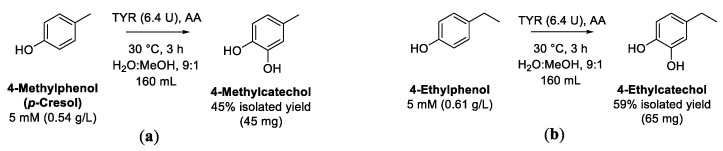
*o*-Hydroxylation of (**a**) 4-methylphenol and (**b**) 4-ethylphenol by tyrosinase (crude extract from *Agaricus bisporus*) [118]. TYR, tyrosinase; AA, L-ascorbic acid (sodium salt).

The hydroxylation of phenols by TYRs yields catechols that are frequently bioactive and much more expensive than phenols. The substrates that can be transformed in this way have recently been briefly summarized [113]. They are, in general, *para*-substituted phenols, and the range of substituents tolerated by tyrosinase is very broad; the substrates are alkylphenols, halophenols, tyrosine, tyrosol, phenylpropanoic acid analogs, etc. [114,115,116,117]. Of these, some alkylphenols (4-methylphenol, 4-ethylphenol) and phenols are lignin-derived compounds. The two alkylphenols are excellent substrates for tyrosinase and can be smoothly converted to the corresponding catechols with catalysts based on tyrosinase from *Agaricus bisporus*. For instance, an inexpensive extract from the fruiting bodies of this fungus was used to prepare 4-methylcatechol and 4-ethylcatechol. The reactions and product purification proceeded to show acceptable isolated yields (Scheme 25) [118]. The two catechol products that also naturally occur in some fermented or smoked foods were found (together with catechol and 4-vinylcatechol) to have beneficial effects on the Nrf2 pathway of cell defense [119].

### 9.2. Oxidation of 4-Ethylphenol to Chiral Secondary Alcohol

**Scheme 26:**
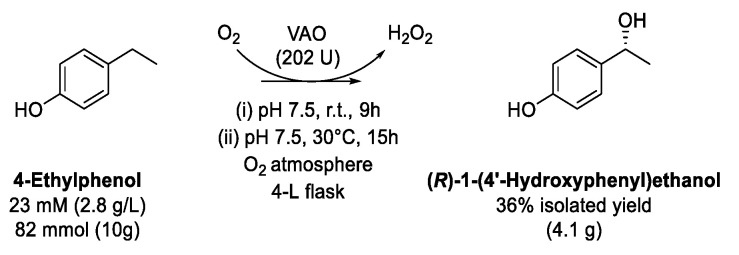
Oxidation of 4-ethylphenol to (*R*)-1-(4′-hydroxyphenyl)ethanol by vanillyl alcohol oxidase (VAO) from *Penicillium simplicissimum*. Cell-free extract from *E. coli* cells producing VAO was used as the catalyst [121].

VAO oxidized not only vanillin alcohol, but also various *p*-substituted phenolics. The enzyme oxidized its substrates via the action of FAD, and the reduced cofactor then reacted with O_2_ to form H_2_O_2_ [120]. In addition, VAO functionalized the aliphatic C-H bond in 4-ethylphenol. Moreover, the reaction proceeded enantioselectively to yield chiral secondary alcohol with an impact on the synthesis of chiral medicines. The reaction was carried out at scales up to 10 g to yield over 4 g of the product [121] (Scheme 26). The investigation of the VAOs’ phylogeny revealed evolutionarily distant VAOs with unexplored biotransformation potential [122].

## 10. Conclusions

Lignin is the largest natural source of aromatic compounds of importance to the chemical industry. The idea of using lignin to produce aromatics is quite old, but is becoming increasingly important in light of the current inadequate supplies and high prices of petroleum. The application of lignin in the chemical industry must overcome the hurdles associated with producing and depolymerizing lignin, upgrading the depolymerization mixtures, isolating individual compounds, and converting them to a broad range of value-added chemicals. These processes are often harsh and environmentally damaging, and consume fossil resources. This compromises the benefits of using renewable materials. Therefore, bio-based methods have been proposed for all steps of lignin valorization. We refer to previous reviews for the use of such methods in lignin separation from (hemi)cellulose and depolymerization. Herein, we have focused on the biotransformations of lignin-derived primary products. The application of bio-based processes for this stage of lignin valorization is relatively straightforward compared to the work-up of technical lignins, as the formeruses monomers.

Our review of the recent literature has shown that promising methods for the production of added-value products (vanillin and its derivatives, syringaldehyde, gallic acid, muconic acids, adipic acid, alkyl catechols, chiral secondary alcohols, etc.) from a few lignin monomers have emerged. Some of the proposed processes demonstrated satisfactory parameters (e.g., selectivity, high conversion), but there are challenges that remain to be resolved, such as the cost of catalyst preparation, side reactions, and difficulties in increasing the substrate concentration due to inhibition by the substrate, product, or intermediate (Table 1). Apart from this, only a few of these processes have been demonstrated with real lignin-based samples, or on a preparative scale.

The processes were proposed either with model substrates or substrates obtained from lignin, mostly as mixtures. The latter often suffered from very low concentrations of the substrates and from unwanted impurities. Generally, the substrate and product concentrations should be increased to make the bioprocesses economical. Improvement of the biocatalysts is necessary in most cases, while the engineered organisms or enzymes and the resulting artificial pathways are probably the most attractive. Although the competition of the purely chemical processes, such as the innovative (Solvay) process to vanillin, is strong, some of the reviewed bioreactions, if optimized, can help lignin-based chemistry to gain more influence on chemical synthesis.

## Figures and Tables

**Table 1:** Comparison of lignin-based biocatalyzed processes for the production of various phenolics.

Product	Substrate	Pros	Cons	Reference
Vanillin	Vanillic acid	Selectivity, high conversion, cofactor recycling	Purified enzyme costs	[44]
	Ferulic acid	Selectivity	Purified enzyme and cofactor costs	[72]
		Selectivity, no cofactors	Purified enzyme costs	[73]
	Isoeugenol	Selectivity, product purity, thermostable catalyst, low-cost substrate, isolated product (1 g)	Inhibition by vanillin ^1^	[107]
Vanillic acid	Ferulic acid	Low-cost substrate (rice bran), crystallized product (4 g)	Non-standard host	[78]
Syringaldehyde	Syringic acid	High conversion, no cofactors	Side product (alcohol)	[60]
Gallic acid	Vanillin	Convergent reactions, use of depolymerized lignin	Moderate concentrations of substrates	
	Vanillic acid			
	Syringic acid			[54]
	*p*-Hydroxybenzoic acid			
	Ferulic acid			
4-Hydroxybenzoic acid	*p-*Coumaric acid	Selectivity, high conversion	Non-standard host; inhibition by substrate	[82]
*cis*,*cis*-Muconic acid	Guaiacol	High conversion; use of depolymerized lignin	Toxicity of catechol (intermediate) ^2^, side product (isomer)	[90,97,99]
Adipic acid	Catechol	High conversion	Moderate concentration of substrate	[97]
	Guaiacol	Acceptable conversion	Guaiacol to catechol reaction rate-limiting	
Methoxy- hydroquinone	Vanillin	Selectivity, acceptable conversion	Moderate concentration of substrate	[51]
	Vanillic acid			
Alkylcatechols	Alkylphenols	Selectivity, high conversion, low-cost substrates, low-cost catalyst, products isolated	Moderate concentration of substrates	[114,118]
(*R*)-1-(4′- Hydroxy-phenyl)ethanol	4-Ethylphenol	Stereoselectivity, low-cost substrate, product isolated (4 g)	Isolated yield moderate, O_2_ atmosphere required	[121]

^1^ Reduced by product extraction [107]. ^2^ Reduced by using the fed-batch mode.
